# Extraction and Analysis of Xylitol in Sugar-Free Gum Samples by GC-MS with Direct Aqueous Injection

**DOI:** 10.1155/2019/1690153

**Published:** 2019-02-07

**Authors:** Suranga M. Rajapaksha, Katherine Gerken, Todd Archer, Patty Lathan, Achala S. Liyanage, Deb Mlsna, Todd E. Mlsna

**Affiliations:** ^1^Department of Chemistry, Mississippi State University, Starkville, MS 39762, USA; ^2^Department of Engineering Technology, Faculty of Technology, University of Sri Jayewardenepura, Gangodawila, Nugegoda, Sri Lanka; ^3^Small Animal Emergency and Critical Care, The Ohio State University, Veterinary Medical Center, Columbus, OH 43210, USA; ^4^College of Veterinary Medicine, Mississippi State, Jackson, MS 39762, USA

## Abstract

Xylitol, a sugar substitute frequently used in sugar-free gum, is generally considered harmless to humans but it can be extremely toxic to dogs. Dog-owning customers are becoming increasingly aware of the risks associated with xylitol-containing chewing gums. However, there remains some uncertainty if these chewing gums are still dangerous to dogs after they have been partially consumed. In this work, a reliable low-cost analytical method has been developed to quantify the xylitol in sugar-free gum samples. Xylitol was extracted from gum samples using water as a solvent. Extractions were analyzed by GC-MS with direct aqueous injection (DAI). This method was successfully applied to over 120 samples including fresh gum and 5 min, 15 min, and 30 min chewed gum samples.

## 1. Introduction

Xylitol can be found naturally in low concentrations in fruits, vegetables, mushrooms, and sugar cane. It is a type of artificial sweetener known as a sugar alcohol [1], commonly used as a reduced calorie sugar substitute in many foods. In food, the primary role of artificial sugar alcohols is to act as sweeteners, but they also influence product texture, preservation, moisture maintenance, and the cooling sensations in the mouth upon consumption [1]. For these many reasons xylitol is extensively utilized in chewing gum, and consumers favor xylitol-containing products because of perceived reduction in energy intake which can produce weight loss [2, 3]. Xylitol is also safe for diabetics because it stimulates much less insulin release than a comparable quantity of table sugar [1]. Xylitol helps to prevent dental caries [4–9], since diet is a major etiological factor in dental health, and limiting the consumption of fermentable carbohydrates and sugars is an effective strategy to control dental caries [10]. Therefore, there is interest in replacing sucrose in chewing gum with nonfermentable sugars such as xylitol, which prevents the lactic acid production that can result in cavities [4–8].

Xylitol has a varied safety margin in mammals. The LD_50_ of xylitol in mice is 20 g of xylitol per kilogram [11] but is nontoxic for both cats and humans. While xylitol consumption has been proven to be beneficial to humans, it can be fatal to dogs. Xylitol is rapidly absorbed in dogs, increasing insulin levels within 15 minutes of ingestion [12]. Hypoglycemia occurs when intracellular transfer of potassium ions is activated by insulin [13]. A dose of 0.1 g of xylitol per kg of dog has been reported to cause hypoglycemia in dogs within 30–60 minutes of ingestion, and some veterinary clinicians have reported liver failure in dogs after 8–12 h [12]. Generally, xylitol ingestion by dogs causes hyperinsulinemia, which can result in vomiting, ataxia, lethargy, weakness, seizures, hypoglycemia, coma, and death [11–16]. Thus, ingestion of xylitol-containing products, such as sugar-free gum, can result in xylitol toxicity in dogs.  Table 1 shows the prediction of minimum number of gum sticks to provide a toxic dose of xylitol (0.1 g of xylitol per kilogram of dog) to cause hypoglycemia [16–18].

Xylitol's presence in chewing gum and other consumer products makes it readily available to dogs with detrimental effects often requiring veterinary care. This study was inspired by many such incidences. Today, many dog-owning customers are aware of the risks associated with xylitol-containing chewing gums. However, there remains some uncertainty as to whether these chewing gums are still dangerous to dogs after they have been partially consumed. The primary goal of this study was to provide conclusive evidence of the toxicity of xylitol-containing chewing gums after partial consumption. Herein, a reliable low-cost analytical method has been developed to quantify the xylitol in sugar-free gum samples.

Numerous methods are commonly employed for the analysis of polyols [19–21] including those that utilize HPLC [22–24], GC-MS [25] with sample derivatization, over-pressured layer chromatography (OPLC) [26], and high performance anion-exchange chromatography (HPAEC) [27]. HPLC seems to be the obvious choice in this analysis; however, separation can be complicated because gum samples often have multiple polyols with overlapping retention times. Consequently, HPLC separation often requires specialty, high-cost columns that may not be available in many analytical laboratories.

A direct aqueous injection GC-MS method utilizing an Agilent 7890A GC/5975C MS has been developed in this study. Direct aqueous injection (DAI) is key to this analysis because polyols are more soluble in water than any other common solvent [28]. Employing DAI with GC-MS analysis has important advantages including high-speed analysis, simplicity, and the elimination of the lengthy sample derivatization steps that are often required [29–31]. Direct aqueous GC injection has been successfully used to analyze polar compounds such as carboxylic acids, ethers, fuel oxygenates, and other fuel components [32–37]. To the best of our knowledge, the method described here is the first to explore the use of GC-MS direct aqueous injection to determine xylitol content of chewing gum samples.

## 2. Materials and Methods

### 2.1. Reagents and Materials

Glycerol (CAS-56-81-5, assay 99.5%, MW 92.09 g mol^−1^), xylitol (CAS-87-99-0, assay 99%, MW 152.15 g mol^−1^), DL-threitol (CAS-7493-90-5, assay 97%, MW 122.12 g mol^−1^), and sorbitol (CAS-50-70-4, assay 99%, MW 182.17 g mol^−1^) were purchased from Sigma-Aldrich. Xylitol-containing Trident sugar-free gum was purchased from Walmart (regular 0.17–0.20 mg of xylitol/piece). DI water was used to prepare all samples and standard stock solutions. Similar weight gum pieces were chosen for analysis.

### 2.2. GC-MS Analysis

An Agilent 7890A/5975C gas chromatography-mass spectrometry (GC-MS) system was used with a water resistant 60 m × 320 *µ*m × 1 *µ*m, 100% dimethylpolysiloxane, Agilent *J&W DB-1* column. [38] The GC oven was programmed to heat as follows: temperature at injection was 216°C, followed by heating from 216 to 230°C at 1°C min^−1^, from 230 to 290°C at 30°C min^−1^, and then holding at 290°C for 3 min. The total program time is 20 min. The carrier gas was He at a pressure of 60 kPa. Using a 10 *µ*L syringe, 1 *µ*L injections were done in split mode (30 : 1) at 280°C. The Agilent 5975C mass spectrometer was operated under scan mode with an electron impact ion source operated at 70 eV. The ion source temperature was 250°C, and the interface temperature was 280°C.

### 2.3. Extraction Method Overview

**Figure 1:**
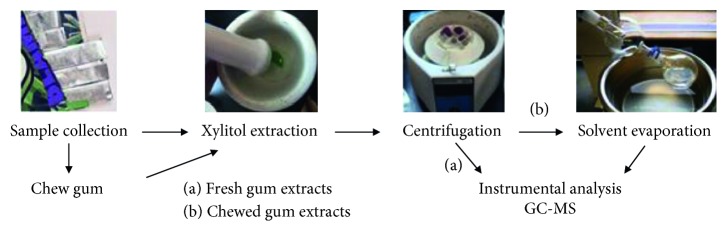
A flow diagram of xylitol analysis method.

A flow diagram of the method is shown in Figure 1 that includes sample collection, xylitol extractions by grinding gum pieces using a mortar and pestle, and centrifugation to remove any particulates before preparing solutions for GC-MS analysis. Since fresh gum samples contain a large amount of xylitol, sample concentration before the analysis was not required (Figure 1, Fresh gum). However, chewed gum samples contain fairly small amounts of xylitol and therefore, extracts must be concentrated for accurate GC-MS analysis (Figure 1, chewed gum). Typically, a nitrogen evaporator is used to concentrate samples. However, in this study, a large amount of water needed to be evaporated which required 2–3 h per sample using the nitrogen evaporator with a bath temperature of 60–70°C. The rotary evaporator proved to be more efficient. With a bath temperature of 40°C, the vacuum (water aspirator) rotary evaporator shortened the concentration step from hours to minutes (maximum 30 min).

### 2.4. Extraction of Xylitol from Fresh Gum Samples

Sample collection: 4 gum packs, each containing 18 gum sticks, were randomly selected from a commercial package containing 14 packs. A total of 12 fresh gum sticks were collected from the gum packs including the 1st, 9th, and 18th gum sticks of each pack to determine xylitol content.

**Figure 2:**
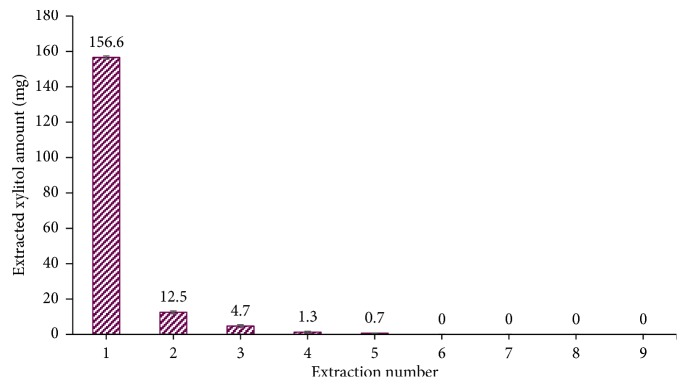
Amount of xylitol extracted after each extraction.

A fresh gum stick was carefully cut into about 6–7 small pieces. Gum pieces were ground for 5 min with 10 mL of DI water. This extraction step was repeated 9 times requiring a total of 90 mL DI water and 45 min to extract xylitol completely from a single gum piece (Figure 2). Nine extractions are recommended to account for differences in an extractor's grinding technique. Gum extracts were centrifuged to remove any particulate, and the supernatant was transferred to a 100 mL volumetric flask. DI water was then added to the 100 mL mark. Exactly 20.0 mL from the gum extraction was transferred to a 50 mL volumetric flask along with 5.0 mL of 5.0 mg mL^−1^ of DL-threitol solution as an internal standard. 1 *µ*L from the solution was injected to the GC inlet. And 10th extraction (10 mL × 5 min) was performed to confirm that no xylitol was still trapped in the gum. This extraction was centrifuged and concentrated into 1 mL before 1 *µ*L was injected for GCMS analysis.

### 2.5. Extraction of Xylitol from 5 min Chewed Gum Samples

Sample collection: 12 volunteers, between 20 and 40 years old, participated in this study. Participants were asked to wash their mouth with water before they chewed a gum stick. Participants chewed their gum pieces with similar starting masses (1.724 g ± 0.008) for 5 min before placing the chewed gum into a sterilized container. Three gum samples from each participant were collected within a 2-day interval (a total of 36 gum samples). The xylitol content of each 5 min chewed gum piece was determined as follows.

The gum stick was ground using a mortar and pestle for 5 min with 10 mL of DI water. This extraction step was repeated *8 times* for a total of 80 mL of DI water and 40 min to extract xylitol completely from the gum piece. The reduced xylitol content in the chewed samples required fewer extraction steps than the fresh gum. Gum extracts were centrifuged to remove any particulate and then the supernatant was carefully transferred to a 100 mL evaporation flask. The pooled extract was concentrated down to 5 mL under reduced pressure using a water aspirated rotary evaporator with a water bath temperature of 40°C. The concentrated sample was carefully transferred into the 10 mL volumetric flask. To ensure complete transfer, the evaporation flask was washed with another 3 mL of DI water. This washing was then transferred to the same 10 mL volumetric flask. 1.0 mL of a 5.0 mg mL^−1^ of DL-threitol solution was added as an internal standard to the flask which was then filled to the 10 mL mark with DI water. 1 *µ*L from the solution was injected to the GC inlet for analysis. And 9th extraction (10 mL × 5 min) was performed using the same gum piece to confirm that no xylitol was still trapped in the gum. This extraction was centrifuged and concentrated into 1 mL, followed by a 1 *µ*L injection into the GC-MS.

### 2.6. Extraction of Xylitol from 15 min Chewed Gum Samples

Sample collection: the same 12 volunteers who participated in the 5 min chewed gum sampling also participated in this study. A similar procedure was used as above. Participants washed their mouth with water before chewing gum pieces with almost identical masses (1.724 g ± 0.008) for 15 min. The chewed gum pieces were collected into sterilized containers before extraction. Three replicates were performed by each participant (total 36 gum samples).

A similar extraction method was used as above with the 5 min samples. However, with these 15 min samples, the 10 mL extraction step was repeated *6 times* with a total of 60 mL of DI water and required 30 min to completely extract xylitol from the gum piece. As with the 5 min samples, the gum extracts were then centrifuged to remove any particulates, and the supernatant was carefully transferred to 100 mL evaporation flask, and water was evaporated to dryness, under reduced pressure using a rotary evaporator. To the evaporation flask, 900 *µ*L of DI water and 100 *µ*L of 5.0 mg mL^−1^ DL-threitol solution were added and mixed. Then, 1 *µ*L of the solution was analyzed by DAI GC-MS. A final 7th extraction (10 mL × 5 min) was performed using the same gum pieces to determine if xylitol remained in the extracted gum pieces. This extract was also centrifuged and concentrated to 1 mL. Then, 1 *µ*L was injected to the GC-MS.

### 2.7. Extraction of Xylitol from 30 min Chewed Gum Samples

Sample collection: the same 12 volunteers who participated for both the 5 and 15 min chewed samples also participated in this study. An identical method was used with the exception being 30 min of chewing before extraction. Xylitol was then extracted and analyzed using an identical method as above for the 15 min samples.

## 3. Results and Discussion

### 3.1. GC-MS Method Development

**Figure 3:**
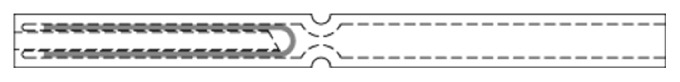
Laminar cup splitter design.

Water is generally considered as a poor solvent in GC analysis due to problems related to backflash and chemical reactivity. Common GC solvents such as hexane, ethyl acetate, acetone, and dichloromethane have vapor-to-liquid volume ratios in the range of 100–300, [38] while the same for the water is found to be 1000. Therefore, 1 *µ*L liquid water injection into the GC liner creates 1000 *µ*L of water vapor [38]. Typical volume of a GC liner is in the range of 200–900 *µ*L. Solvent vapors that expand to exceed the volume of the GC liner results in backflash which can cause severe separation and liner problems. The use of a laminar cup splitter (Figure 3) is suitable for such situations when injecting large volumes of low volatile compounds. With a laminar cup inlet, the injected liquid will be trapped at the GC liner base until all the liquid completes vaporization. Chemical damage to the stationary phase is another problem related to GC water injection. However, immobilized and cross-linked nonpolar liquid film columns are found to be stable towards GC water injections [39]. Maintaining a stable vacuum can also be a concern with GC water injections. Therefore, high capacity pumps were used to obtain best results [40]. In order to avoid damaging the stationary phase and allowing high temperature analysis, a water resistant, 100% dimethylpolysiloxane, low-bleed, cross-linked, and water rinsable column was used in this study.

**Figure 4:**
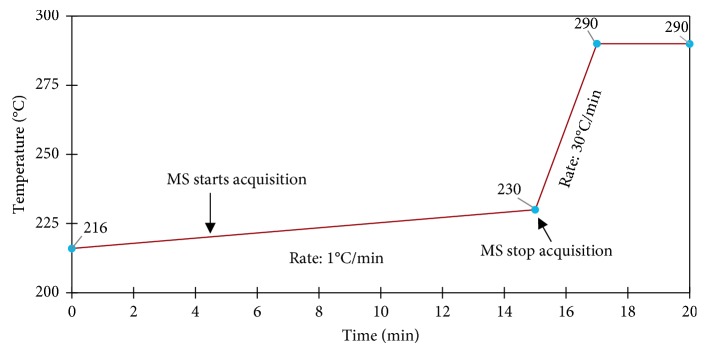
GC oven program.

Analysis of xylitol (boiling point 216°C) in a complex mixture requires both high temperature injection and temperature programming to ensure chromatographic separation. The GC oven was programmed with an initial temperature at the boiling point of xylitol to facilitate high temperature analysis, while reducing column bleed and improving limits of detection (Figure 4). The mass detector was programmed to analyze mass fragments between 35 and 350 *m/z* from 4.5 min to 15 min in order to avoid detector saturation caused by volatile components of gum extracts which elute before 4.5 min. After 15 min, the GC was baked out for 5 min (a 2 min heating ramp from 230°C to 290°C followed by holding for 3 min at 290°C) to remove residual sorbitol still trapped in the column. Following the fresh gum (high xylitol concentration) analysis, a blank run was performed to confirm no sorbitol remained trapped in the GC system before further analysis.

**Figure 5:**
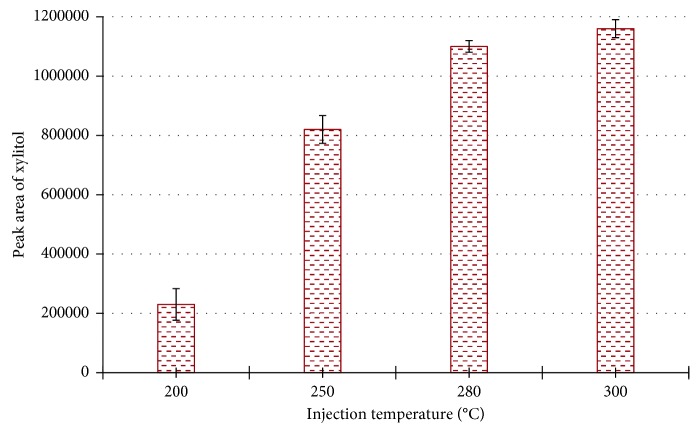
Effect of injection temperature on xylitol peak area. Note: error bars indicate standard deviation (*n* = 3).

The effect of injection temperature was studied to facilitate complete vaporization of xylitol in the GC inlet. Raising the injection temperature from 200°C to 280°C increased xylitol peak area by a factor of 5. However, only a 5% increase was observed when the inlet temperature was increased from 280°C to 300°C (Figure 5). Operating the inlet at 300°C may accelerate septum failure; therefore, 280°C was selected as the optimum injection temperature.

### 3.2. Selection of Internal Standard

**Figure 6:**
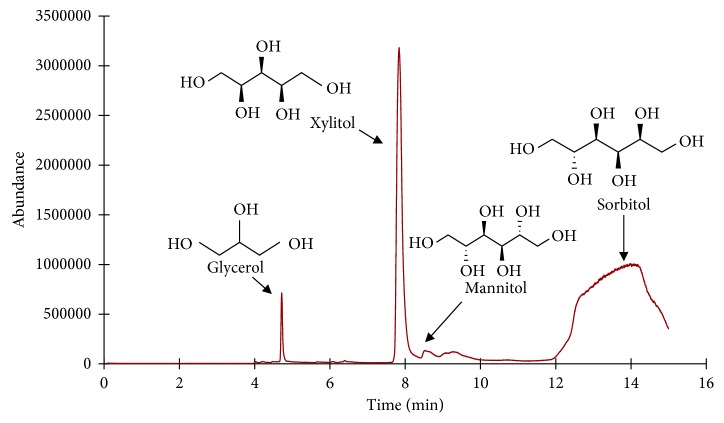
Total ion chromatogram (TIC) of Trident gum extract.

Choosing a correct internal standard (IS) can improve a method's accuracy and precision. Method development for GC-MS often utilizes an internal standard to account for routine variation of the instrument response and injection volumes. An internal standard should be chemically similar to the analyte but it should not be naturally present in any of the samples to be analyzed. A Trident spearmint flavor gum piece contains three polyols in large quantities: xylitol, glycerol, and sorbitol. Mannitol is also present but at low concentrations compared to the xylitol and sorbitol content of the gum piece (Figure 6).

**Figure 7:**
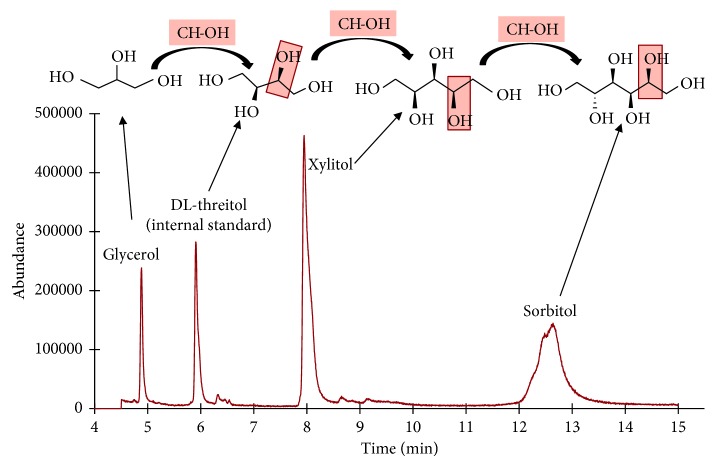
Total ion chromatogram (TIC) of Trident gum extract with internal standard.

Various chemicals were tested as an internal standard for this study including ethylene glycol, 3,5-dimethoxyphenol, 2-methoxyphenol, terpineol, 2-nonanol, and DL-threitol. Both 2-methoxyphenol and DL-threitol were found to be suitable for analysis in the terms of retention times, since they did not overlap with any peaks of the Trident gum extract. However, DL-threitol was picked as the better internal standard because it has the same functional groups as xylitol. More importantly, glycerol, threitol, xylitol, and sorbitol are members of a series of sugar alcohols where the difference between any two compounds in a sequence varies by one carbon atom, two hydrogen atoms, and one oxygen atom, as CH–OH unit (Figure 7). Therefore, DL-threitol is a suitable internal standard in this analysis.

### 3.3. Recovery

Recovery tests were performed to evaluate extraction efficiency. Gum base left after the 10th extraction was spiked with a known amount of solid xylitol (180.0 mg). The xylitol was thoroughly ground into the gum base using a mortar and pestle before extraction and DAI GC-MS analysis using the fresh gum method described above. This procedure was repeated for five gum-base samples (GB-1 through GB-5). Recovery values (Table 2) using the fresh gum extraction method were 95–99% with relative standard deviations of less than 1%. Acceptable recovery values and low standard deviations indicate high accuracy and precision of the method; thus reliable quantification is expected using this method.

### 3.4. Detection Limit

Limit of detection (LOD) and limit of quantification (LOQ) were determined. An internal standard calibration graph was plotted using the ratio of peak areas of the internal standard (DL-threitol) versus the concentration of xylitol. Good linearity was observed, with a square of the correlation coefficient (*r*^2^) of 0.9995 in the range of 0.7 to 2.0 mg mL^−1^ xylitol. A signal-to-noise approach was selected to determine limits of detection and quantification. LOD was determined to be 0.1 mg/mL using a signal-to-noise (*S*/*N*) ratio of 3, while the LOQ was determined to be 0.7 mg/mL using a S/N approximately of 10.

### 3.5. Statistical Analysis

Analysis of variance with Tukey's honestly significant difference test (*P* < 0.05, IBM SPSS Statistics, Version 24) was performed to determine the significant differences in xylitol contents in fresh and chewed gums.

### 3.6. Determination Effect of Chewing Rate on Xylitol Release from Gum Base

**Figure 8:**
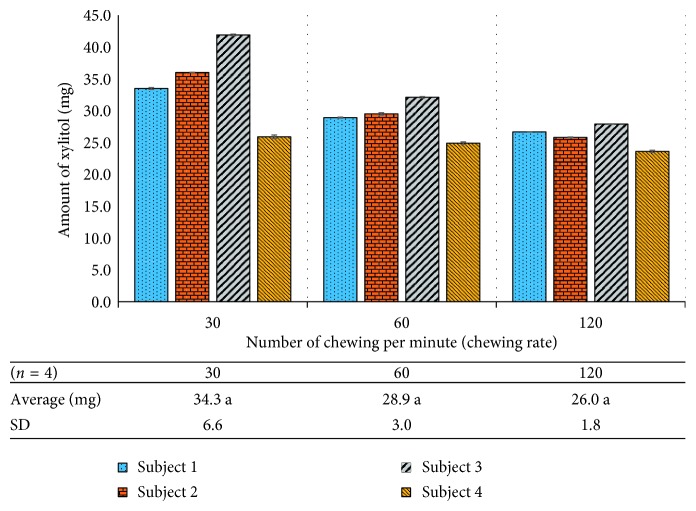
Amount of xylitol remaining in a gum piece chewed by four participants at three different rates (numbers sharing similar lower case letters indicate no significant difference).

Effect of chewing rate on xylitol release was studied. Four volunteers were randomly selected for this experiment. Each participant was given three gum sticks with similar masses (1.724 g ± 0.008). Chewing rates were selected after considering general chewing habits. We observed that a person starts chewing a gum piece at a rate between 30 and 120 chews per minute for the first two minutes due to the sugar taste. Chewing rates then decrease with time due to decreased sugar content of the gum. Three chewing rates 30, 60, and 120 chews per minute were selected. Participants were asked to chew gum pieces at the selected rates for 2 minutes. The method, which was used for the fresh gum analysis, was used to determine the xylitol content of chewed gum samples in this experiment (Figure 8).

Results show that chewing rate had no significant effect on the remaining xylitol content in the 2 min chewed gums (*P* < 0.05). It should be noted that there appears to be a trend that more xylitol is released with more rapid chewing; however, a larger sample size is required for proof.

### 3.7. Sample Analysis

Trident spearmint flavor gum pieces were selected for this study. In order to determine the range of xylitol content in a gum stick, 4 gum packs were chosen randomly, each with 18 gum sticks. The 1st, 9th, and 18th sticks of each pack were selected for analysis. The amount of xylitol in the fresh gum pieces were determined using the fresh gum analysis method established above. Each sample was analyzed by DAI GC-MS three times, and the results are summarized in Table 3 (P1–P4 represent gum pack number, ST 1, 9, and 18 represent gum stick number). The xylitol content of the fresh gum samples ranged from 170.7 to 193.0 mg with an average of 179.2 mg of xylitol per piece. There were no significant differences in the xylitol content of the fresh gum packs used in this study (*P* < 0.05).

**Figure 9:**
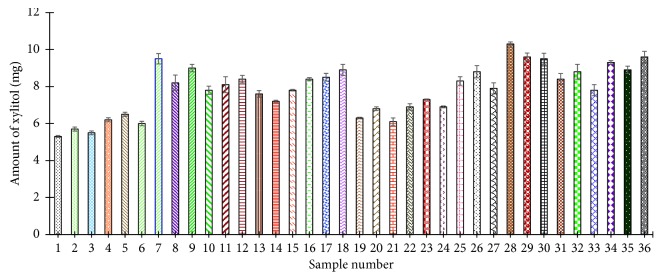
Determination of xylitol content in 5 min chewed gum samples.

A total of 36 samples (3 each for the 12 participants) were analyzed to determine the amount of xylitol in 5 min chewed gum samples, and the results are summarized in Figure 9. The mass of xylitol ranged from 5.3 to 10.3 mg with an average of 7.8 mg per piece. On average, the 5 min chewed gum samples retain about 4% of the original xylitol in a fresh gum stick.

**Figure 10:**
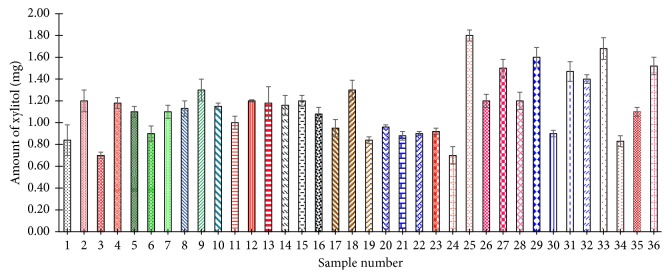
Determination of xylitol content in 15 min chewed gum samples.

A total of 36 (3 from each participant) of the 15 min chewed gum samples were also analyzed (Figure 10). The amount of xylitol found in these samples ranged from 0.7 mg to 1.8 mg with an average of 1.1 mg per piece. This represents about 0.6% of the original xylitol. As shown in Figure 10, the amount of xylitol remaining in gum after 15 min of chewing varies significantly from person to person (*P* < 0.05).

There was no xylitol above the LOD in any of the 36 samples collected after a 30 min chewing period. Approximately 99.4% of xylitol is removed from the gum within the first 15 min, and another 15 min of chewing time reduced the xylitol content below the method limits to detect.

Table 4 was generated to demonstrate the number of gum sticks (fresh, 5 and 15 min chewed) required to deliver a toxic dose to each dog size resulting in hypoglycemia. Although a large number of 5 and 15 min chewed gum pieces are required to cause hyperglycemia in a 2 kg Chihuahua, chewed gum samples should not be neglected because they still pose a threat, particularly to smaller dogs. In general, a dog's size affects its tolerance to xylitol poisoning: the smaller the dog, the less tolerant and vice versa. While a large dog may have little to fear after eating 5 min chewed samples, a small dog is very much at risk to the dangers of ingesting xylitol. Eating several 30 min chewed gum sticks are not likely to result in xylitol poisoning; however, digestion issues could occur.

## 4. Conclusions

A method for the analysis of xylitol in sugar-free gum was successfully developed using GC-MS with direct aqueous injection (DAI). Additional cleanup steps and sample derivatization were not required for the analysis, resulting in short analysis times. Spiked recoveries of the sample ranged from 95 to 99% while RSD ranged from 0.17 to 0.72% (*n* = 3) supporting that this is a reliable quantification method, with high accuracy and precision. Thus, this method possesses advantages in terms of efficiency, selectivity, and accuracy.

## Figures and Tables

**Table 1:** The amount of xylitol and number of fresh gum sticks that can cause hypoglycemia in dogs [16–18].

Size of the dog	Amount of xylitol to cause hypoglycemia in dog (g) (dose: 0.1 g of xylitol per kg of dog)	Required number of gum sticks		
		Ice breakers (1.5 g of xylitol/gum stick)	Stride (0.2 g of xylitol/gum stick)	Trident (0.2 g of xylitol/gum stick)
2 kg Chihuahua	0.2	1	1	1
4 kg Yorkie	0.4	1	2	2
6 kg Jack Russell Terrier	0.6	1	3	3
12 kg Border Collie	1.2	1	6	6
25 kg Golden Retriever	2.5	2	12	12

**Table 2:** Precision and recovery of fresh gum analysis method (*n* = 3).

Gum base no.	Spiked (mg)	Measured (mg)	Recovery (%)	RSD (%)
GB-1	178.2	177.2	99.4	0.31
GB-2	180.3	178.4	98.9	0.18
GB-3	179.4	177.2	98.8	0.17
GB-4	180.6	176.6	97.8	0.24
GB-5	178.4	169.9	95.1	0.72

**Table 3:** Determination of the xylitol content of Trident spearmint flavor gum (regular care).

Gum piece ID	Xylitol content (mg)	SD (*n*=3)	Gum piece ID	Xylitol content (mg)	SD (*n*=3)
P1-ST1	180.8	0.8	P3-ST1	187.8	0.5
P1-ST9	179.1	0.2	P3-ST9	179.8	0.6
P1-ST18	179.7	0.3	P3-ST18	171.3	0.2
P2-ST1	193.0	0.3	P4-ST1	170.7	0.4
P2-ST9	187.8	0.6	P4-ST9	172.4	0.4
P2-ST18	174.0	0.4	P4-ST18	181.1	0.2

**Table 4:** Amount of gum sticks required to supply toxic dose to make a dog sick.

Size of the dog	Amount of xylitol to cause hypoglycemia (g)	Number of gum pieces required to supply toxic dose (dose: 0.1 g of xylitol per kg of dog)		
		Fresh (xylitol = 179 mg)	5 min chewed (xylitol = 7.8 mg)	15 min chewed (xylitol = 1.1 mg)
2 kg Chihuahua	0.2	1	26	182
4 kg Yorkie	0.4	2	51	364
6 kg Jack Russell Terrier	0.6	3	77	545
12 kg Border Collie	1.2	7	154	1091
25 kg Golden Retriever	2.5	14	320	2273

## Data Availability

The data used to support the findings of this study are included within the article.
